# Human Factors and Human-Computer Considerations in Teleradiology and Telepathology

**DOI:** 10.3390/healthcare2010094

**Published:** 2014-02-19

**Authors:** Elizabeth A. Krupinski

**Affiliations:** Department of Medical Imaging & Arizona Telemedicine Program, University of Arizona, 1609 N Warren Bldg 211, Tucson, AZ 85724, USA; E-Mail: krupinski@radiology.arizona.edu; Tel.: +1-520-626-4498; Fax: +1-520-626-4376

**Keywords:** teleradiology, telepathology, workstations, displays, human factors

## Abstract

Radiology and pathology are unique among other clinical specialties that incorporate telemedicine technologies into clinical practice, as, for the most part in traditional practice, there are few or no direct patient encounters. The majority of teleradiology and telepathology involves viewing images, which is exactly what occurs without the “tele” component. The images used are generally quite large, require dedicated displays and software for viewing, and present challenges to the clinician who must navigate through the presented data to render a diagnostic decision or interpretation. This digital viewing environment is very different from the more traditional reading environment (*i.e.*, film and microscopy), necessitating a new look at how to optimize reading environments and address human factors issues. This paper will review some of the key components that need to be optimized for effective and efficient practice of teleradiology and telepathology using traditional workstations as well as some of the newer mobile viewing applications.

## 1. Introduction

Over the past 25 to 30 years radiology has changed dramatically as advances and improvements in imaging acquisition devices and telecommunications have occurred. Additionally, public expectations in response to these changes have changed, contributing to increased radiologist sub-specialization [1,2]. A significant consequence of this sub-specialization is increased utilization and growth of teleradiology, as referring clinicians and patients expect and often demand expert interpretation of images not only in major urban areas, but also in areas that are rural and medically underserved. Thus, teleradiology is perhaps the most successful and well-established telemedicine service, with over 70% of radiology practices in the United States (U.S.) using on-call emergency reporting, and general teleradiology being provided regularly by Nighthawk services around the world [2].

One consequence of the demand for imaging and sub-specialty interpretation is that radiologists more than ever are expected to provide service 24/7, requiring residents to be on-call after hours and on weekends, and expert attendings to be more available. Teleradiology helps makes this possible, and professional societies such as The American College of Radiology (ACR) [1] and The European Society for Radiology (ESR) [2] encourage radiologists to use teleradiology by incorporating general and sub-specialist radiology services where traditional on-site radiology services and expertise are lacking. Further promoting adoption of teleradiology, the ACR recently updated their teleradiology guidelines noting that continued development of protocols and software to enable bidirectional communication between physicians, technologists, and imaging managers is required; as are better protocols for electronic medical record integration, peer review interfaces, and dictation systems that eliminate manual interfaces (e.g., paper-based tools, non-voice activated/controlled dictation systems) [1].

In pathology, there have been significant changes over the past 30 years as well in the types of technologies used to acquire images from glass slides, and the future will bring even more changes [3,4,5,6,7,8]. Advances in image analysis and processing techniques for providing interpretation aids to the pathologist using whole slide images (WSI) as well as in developing automated image analysis tools are also occurring at an astounding pace [9,10,11,12,13,14]. There is also increasing demand for technical standards [15,16,17,18,19] for WSI acquisition and display, and there are already clinical practice guidelines for telepathology [20,21,22]. Although telepathology has not quite achieved the widespread success that teleradiology has [23,24,25,26], it is increasingly being used clinically as well as for educational and administrative purposes [27,28,29].

A recent survey of Canadian pathologists revealed some interesting attitudes pathologists have regarding telepathology [27]. They found that telepathology is used in about 43% of institutions, especially for teaching (65%) and operating room consults (46%). About 3% use telepathology for consult services, 15% for routine diagnoses, and 17% for quality assurance. About 71% of the respondents believe there is a need for telepathology and 85% use digital images in practice. The most preferred applications are teaching and consultation, and the main advantage is easier access to cases. The respondents also noted that the main limitations are cost and image/diagnostic quality.

Regardless of how medical images are acquired or processed, the information contained in them is not self-explanatory and therefore needs to be interpreted. Thus, the display and optimal visualization of image data is critical since human observers are required to perform this interpretation. This paper will review some of the key components that need to be optimized for effective and efficient practice of teleradiology and telepathology using traditional workstations as well as some of the newer mobile viewing applications.

In the case of radiology, a traditional reading environment refers to viewing film (hardcopy) images using a light box or alternator as opposed to viewing digitally acquitted images on a computer (or other electronic) display. Although the latter is the norm in many institutions, there are still numerous clinics and other facilities in the U.S. and around the world that are still film based. In pathology the traditional environment refers to viewing glass slides through a light microscope, while digital or whole slide imaging refers to scanning glass slides into a digital format (using a variety of optical techniques) and viewing the acquired images on a computer or other display. In both specialties, this transition into the digital environment has generally necessitated renovating reading rooms to accommodate digital displays, computers, desktop space for a mouse, and electronic dictation systems; as well as new lighting, noise reduction, and desk and chair choices.

## 2. Medical Image Displays and Perceptual Processes Underlying Image Interpretation

Viewing and interpretation are at the core of medical image interpretation [30,31], and it is important to consider them from two perspectives [32]. First is the display technology [33,34], and the ways that variables such as luminance, spatial noise and color impact image quality and the visibility of diagnostic information needed to render a decision. Second is the human observer (*i.e.*, pathologist, radiologist) who relies on her/his perceptual and cognitive systems to accurately and efficiently process image data and information. In this respect, it is useful to understand some basic properties of the human eye-brain system.

### 2.1. Perception Basics

It is not necessary to fully understand the anatomy and physiology of the human visual system to appreciate what it is capable of (or limited by), although there are a few very relevant properties with respect to displays and medical images. Photoreception is the means by which light from the environment creates changes in the photoreceptors or nerve cells in the retina (approximately 115 million rods and 6.5 million cones) [35]. The rods, which are located mostly in the retina periphery, are used to sense contrast, brightness, and motion. The cones, located in the foveal and parafoveal regions, are used for fine spatial resolution, spatial resolution and color vision. When hit by light, specialized pigments in the rods and cones undergo chemical transformations, and light energy is converted into electrical energy that acts on nerve cells connecting the eye to the optic nerve and subsequent visual pathways extending to the visual cortices in the brain.

**Figure 1:**
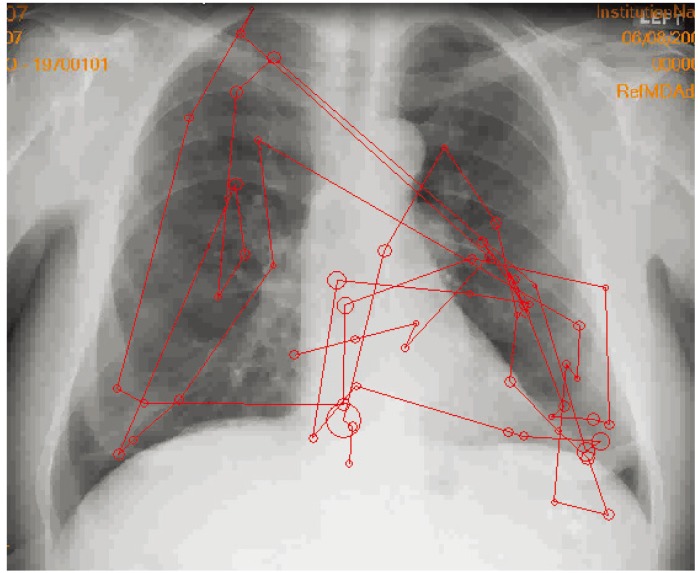
Example of how a typical radiologist examines a chest image. Circles represent fixations or where the eyes land with foveal vision and the size reflects how much time is spent fixating each location with larger circles indicating more time. Lines are saccades or jumps the eyes make between fixations.

Especially important for viewing radiology and pathology images is spatial resolution or the ability to see fine details. It is highest at the fovea and declines sharply towards the peripheral retina. What this means is that to see image details pathologists and radiologists must either sequentially move the image (pan) so that every part is placed in front of the fovea or, more practically, they must move the eyes (*i.e.*, fovea) around the image (Figure 1).

The fact that images must be searched impacts the way they are displayed, and for the most part it is common in both specialties to initially display the entire image all at once at the highest resolution possible then provide tools (*i.e.*, zoom and pan) to navigate through the image at higher resolutions as suspicious or informative image details attract attention.

### 2.2. Implications for Choosing Displays

From a human factors perspective there are a couple of important considerations with respect to displays and spatial resolution. One consideration is display size and as already noted both pathology and radiology images are quite large and should be displayed at as high a resolution as possible when viewing the entire image but even then users need to scan the images. In pathology the typical WSI is full color (24 bits per pixel) with dimensions many thousands of pixels in width and height depending on the scanner used to acquire the image, and a case may contain multiple images resulting in hundreds to thousands of megabytes per case. In radiology, mammography is the highest resolution image with some systems having 50-micron pixel detectors which results ni images with greater than 5,000 pixels in each direction and even with compression the result is files bigger than 60 MegaBytes. The physical size displayed on the monitor varies of course depending on the monitor itself, but the market is promoting both monochrome and color 10 MegaPixel displays in medical imaging with 30 inch and greater screen sizes!

Radiology has established minimal display requirements [36], but the only pathology guideline is in the Canadian document [21] which says that monitors must be of adequate size and resolution to provide necessary details for interpretation. In general, when the diagonal display distance is about 80% of the viewing distance then entire scene can be well visualized [36]. Most viewers sit about 60 cm from a display corresponding to a 53 cm diagonal display size—or about 21” which is a fairly common display size. 

Display size however is not the full story—the pixel size needs to be taken into account as well. Pixel pitch or the spacing between pixel structures determines the amount of detail that can be presented. At about 60 cm viewing distance the human visual system is capable of perceiving spatial frequencies up to about 2.5 cycles/mm, so a pixel pitch 0.200–0.210 mm is recommended so that images details are visible but the individual pixels and their structures are no visible [36]. In general, a 1,500 × 2,000 display with a pixel pitch of 0.210 will have a 52.5 cm diagonal and should be suitable for displaying most medical images.

Contrast resolution is the ability to distinguish differences in intensity in an image, allowing one to distinguish between objects and background. Display contrast resolution is a function of its luminance levels. In general, the lower the minimum and higher the maximum luminance are the better the contrast ratio. A minimum luminance of 0.8 cd/m^2^ and maximum of at least 350 cd/m^2^ are adequate for viewing most medical images although higher maximum levels are generally preferred and available even on most high-end commercial (non-medical grade) displays.

## 3. Display Calibration

### 3.1. Grayscale Calibration

Calibrating displays is another critical issue. In radiology it is recommended that the Digital Imaging and Communications in Medicine (DICOM) grayscale display function (GSDF) be used for all diagnostic displays [37]. The DICOM GSDF maps image pixel values to a specific displayed luminance, describing that way to calibrate a display to improve contrast resolution. For a standard contrast range, displays calibrated to the GSDF will show consistent grayscale presentation of images even if they are shown on different displays, and studies have shown that observer performance is improved when displays are calibrated to the GSDF than to an uncalibrated display or one calibrated with a method not optimized for grayscale medical images [38].

### 3.2. Color Calibration

Calibration of color displays for telepathology WSI is a very different story. With WSI this is quite important as the variety of displays available and used for viewing WSI is quite large (ranging from high-end medical-grade to commercial off-the-shelf (COTS) low-end displays), and there are few if any regulations or practice guidelines for display performance specifications such as minimum resolution, bit-depth, minimum/maximum luminance, white point, color temperature or calibration to control progressive degradation. Pathology samples are stained with a wide variety of colored dyes. The problem is exacerbated by the fact that digital pathology highlights the color differences between samples, laboratories and scanning and display systems and these often very noticeable color differences may affect diagnostic accuracy as well as the results of image analysis algorithms. In an effort to address these issues (as well as color issues in other medical imaging applications), there was a “Summit on Color in Medical Imaging” hosted by the Food and Drug Administration (FDA) and International Color Consortium (ICC) in May 2013 [19]. Efforts are underway by participants and other researchers to develop a standard display function for medical color imaging. There are older recommendations such as using the Macbeth Gretag color checker [39], but this method is generally based on visual matching of colors on a reference chart with those on the display (although a photometer can be used) and this is rather subjective. Objective measures for any calibration are preferred.

There are very few studies on whether calibrating color displays impacts diagnostic performance, but one study has shown that accuracy is worse without calibration and viewing times are significantly longer. From a human factors perspective, anything that reduces user efficiency is unacceptable (unless it has a profound increase on decision quality); and in today’s medical environment where there are increased demands to see more patients and read more images increased viewing times are unacceptable. 

In the one study that did examine performance as a function of whether the color display was calibrated or not [40], a set of 250 breast biopsy virtual slide regions of interest (half malignant, half benign) were shown to six pathologists, once using a calibration protocol [41] and once using the same display in its “native” off-the-shelf uncalibrated state. Diagnostic accuracy and time to render a decision were measured. Performance was measured using Receiver Operating Characteristic (ROC) techniques using Az (area under the curve) as the performance metric. With the calibrated display Az was 0.8570 and with the uncalibrated display it was 0.8488 (*p* = 0.4112). In terms of interpretation speed, mean time with the calibrated display was 4.895 s and with the uncalibrated display was 6.304 s which was statistically significant (*p* = 0.0460). The results suggest a slight advantage diagnostically for a properly calibrated and color-managed display and a significant potential advantage in terms of improved workflow. 

### 3.3. Calibration Tools

There are a number of display companies today that include calibration software and often integrated photometers with their medical-grade displays. Quite often they also provide remote monitoring services that include automatic recalibration of displays as they drift from ideal with age. In many large departments this is a very reasonable option, especially if there are a lot of displays required and a small or infrequent team of IT (information technology) staff available to perform regular calibrations. The service of course usually comes with a price tag. There are however a number of calibration tools that can be purchased (although they do differ in quality so it is worth perhaps conferring with a medical physicist or someone experienced in calibration prior to purchasing) and used. The key to this option is making sure the tools are used and used properly. Non-medical displays tend to age faster and drift off calibration faster than medical-grade displays so they need to be monitored and calibrated more often (probably once a month). Busy clinicians often forget or simply neglect to incorporate regular calibration into their routine. Laptops can be calibrated with these tools as well, although being mobile the ambient light conditions can vary considerably making it necessary to calibrate more often is the viewing environment changes. Truly mobile devices like SmartPhones are more difficult if not impossible to calibrate so care should be taken with these types of devices for primary interpretations.

## 4. Navigating Through Images

As already noted, radiologists and pathologists need to search through displayed images to find and interpret relevant image features for diagnoses. From a human factors perspective it is important to understand how this search occurs so we can understand how different display parameters and interfaces impact diagnostic accuracy and interpretation efficiency. Eye-tracking studies are useful in assessing observer performance in terms of three main parameters: the time it takes the observer to first fixate (*i.e.*, have the eyes land on) diagnostically relevant areas of interest; the total time the observer looks directly at these locations; and the total viewing time spent on the entire image. In general, correct decisions and efficient search are characterized by shorter time to first fixate, less time spent rendering a decision, and less total viewing time.

### Visual Search

In 1963, Llewellyn-Thomas and Lansdown [42] conducted the first reported eye-position study in medical imaging using a set of radiographic images. They showed that search patterns are unique to the individual and tend to be non-uniform in coverage. Since then numerous studies in radiology have examined aspects of search such as why errors occur (false negatives and false positives) [43,44,45,46], how experts differ from novices [47,48,49], and how different display parameters affect diagnostic accuracy and visual search efficiency [50,51].

In pathology Tiersma *et al.* [52] found that pathologists viewing WSI generally adopt search strategies similar to those of radiologists. They found that readers are attracted to some slide/specimen locations for relatively short periods of time and to others for longer periods of time, and areas examined for longer periods of time generally have more relevant diagnostic information. Roa-Pena [53] found (as did [44,54]) that areas looked at for extended periods of time are often relatively common between readers-regions of interest with diagnostic information attract attention across readers.

Some of the differences observed in search patterns between expert and novice pathologists and radiologists are not only interesting but have human factors implications as well. Key indications of expert interpretation of medical images are consistent, accurate and efficient diagnostic performance, which require not only dedicated training and experience but some degree of talent, aptitude and motivation. Experts are better able to search, process, and interpret larger perceptual units than those with less skill because they are better able to recognize these units more efficiently and effectively as configurations or chunks of information rather than individual pieces [55,56,57,58,59,60,61,62]. The mechanism behind this phenomenon is thought to be due to perceptual and/or cognitive tuning to the visual task as the observer encounters more exemplars over time with suitable training and feedback.

As noted earlier, pathology and radiology images are generally quite large and the displays available often cannot display the full images at full resolution. This is especially true when commercial (*i.e.*, non-medical grade) displays are used. The fact that the entire image may not be viewed at full matrix size is not an especially troublesome issue since zoom and pan are nearly always available with most viewing software programs but if zoom is not used diagnostic accuracy could be impaired [63]. With respect to human factors, the way that radiologists and pathologists scan full *vs.* zoomed images can impact the way that user interfaces are designed. For example, in one eye-tracking study it was found that at low magnification cytologists view about 65% of the slide and as magnification increases scanning becomes much more focused on specific image details [62]. Perhaps with automated analyses of image content that discriminates between relevant and irrelevant slide content, it might be feasible to automatically tailor the way that image data are presented to the pathologist, zooming and panning to the most relevant areas for diagnostic interpretation [13,14].

## 5. Reading Environment

### 5.1. Understanding Workflow

In order to optimize the reading environment it is important to understand what the reading process and workflow consists of [64,65,66,67,68]. For example, Randell *et al.* [66] studied four gastrointestinal histopathologists regarding the range of activities engaged in when viewing slides to determine how to best set up a digital viewing environment. They video-taped 41 cases and found not unexpectedly that mean duration (7 min) varied for each case (range 1–56 min) but overall was fairly consistent. About 66% of the time was spent viewing slides followed by 22% dictating findings. The rest of the time they did paperwork (13%), were on the computer (6%), made notes (5%), annotated slides (3%), referred to a book (2%) and filled in forms (2%). Pathologists clearly multi-task and do more than just read and report out cases. Ho *et al.* [68] found that although pathologists typically engage in lots of tasks, the one that involves the most steps in anatomic pathology workflow is “make and deliver a diagnosis”. In the digital environment it is the main task as well and thus optimizing the display and reading environment is critical.

An interesting examination of teleradiology operations [69] looked at the various ways that teleradiology workflows occur within the context of an existing PACS (Picture Archiving and Communications System)—which is how most practices developed. In this situation, attending radiologists use teleradiology for remote site access and they typically share relevant patient data, meaning that to some extent they need to at least be aware of the remote site's workflow. This can get cumbersome and take time especially when radiologists serve multi-sites, that require different remote access and security protocols. Using simulation techniques, they found that the least efficient workflow is typically with teleradiology companies that read for multiple facilities, since they typically employ off-site radiologists that do not share the workflow of the sites they serve. They suggest that a “super-PACS” can increase efficiency by enabling all users, regardless of location, to work locally and fully participate in the workflow of every site sites that use their own radiologists to perform all radiology tasks including interpretation of off-hour examinations. A SuperPACS uses a global work list, global access to imaging and supporting studies, one virtual desktop viewer, global access for referring physicians, and workflow optimization and monitoring tools. These technologies would allow a radiology group to serve multiple sites with disparate PACS, RIS (Radiology Information System), reporting and other IT systems. Radiologists can participate in the reading workflow of each site regardless of their physical location.

### 5.2. Input Devices

Once the overall workflow has been characterized, there are a number of human factors issues that are important to consider at a more individual user level. The type of input device someone uses is generally a personal preference and common options include keyboards (typically with hot key options), mouse (with or without a scroll wheel and with various number and types of buttons), and track balls. The input device used is a function of user comfort since it is going to be used for every case all day, and as with any other computer interaction the task is repetitive and continuous. Users should be aware of the risk of carpal tunnel syndrome and other repetitive musculoskeletal injuries since this is not insignificant [70]. If someone chooses to use a device other than the mouse they should know that it will generally require a learning phase so there may be a short period in which workflow slows down a bit before it speeds up with familiarity.

There have been a number of studies in radiology and pathology on alternative input devices that have demonstrated faster interactions, fewer steps, *etc.*, but surprisingly most users continue to use the mouse—perhaps out of simple familiarity. For example, Yagi *et al.* developed a prototype WSI viewer using a PlayStation3 with wireless controllers [71]. They found that it took very little training for users to get comfortable with it and most users were quite satisfied with the functionality and speed. In radiology, similar studies have been done with PlayStation controllers, joysticks and other interfaces, all with similar results [72,73]. With a little bit of training other devices are readily adaptable to viewing medical images—most commercial medical workstations simply rarely offer them.

### 5.3. Other Environmental Considerations

Ambient lighting is often ignored as most people are used to using computers in a variety of environments. For medical imaging, however, it is recommended that 20–40 lux of ambient light be used since this will avoid most reflections and glare on the display and still provide adequate lighting for the human visual system to adapt to the surrounding environment and the displays [36,74]. If feasible, ambient lighting should be indirect and backlight incandescent lights with dimmer switches rather than fluorescent are recommended. As incandescent lights are being phased out and compact fluorescent lamps (CFLs) are replacing them, it is important to note that CFLs have longer warm up times before reaching maximum brightness so image reading should be delayed until ambient lights are stable. It is also important to remember that lab coats and light colored clothing increase reflections and glare even with today’s displays so they should be avoided if possible.

Other environmental issues include how much heat and noise does a workstation produce? If a workstation produces too much heat it may be necessary to improve airflow both for the computer and the user. Most computer systems are pretty quiet, but high-performance workstations often use fan-cooled systems that produce noise levels that might be distracting. Overall noise can be an issue and efforts should be made to reduce it as much as possible, including “noise” generated by other clinicians in the room dictating reports or holding conferences. An easy way to reduce noise is to install noise reducing ceiling tiles and carpet, and using cloth covered room separators between workstations (slightly taller than the workstation area to promote privacy but not to the ceiling so that airflow is maintained). If noise is still a problem after such measures have been taken, noise cancelling headphones might considered or even piping white noise into the reading room can help mask sounds.

### 5.4. Software Tools and Image Presentation

Image processing and manipulation tools are also very important from a human factors perspective. Both radiology and pathology utilize a wide variety of tools from simple window/level and zoom/pan manipulations to more complex image processing and analysis. Users should be able to use the basic navigation tools without any training (or very minimal) and without much prior exposure. Systems and interfaces should be user friendly and easy to customize. Simple menus and file managers, single mouse click navigation, visually comfortable colors or gray scales and an uncluttered workspace are all recommended. Customization means that images should be easily adjustable to meet personal visual preferences and interpretation patterns plus easy restoration of default values. 

From a perceptual point of view, the default image presentation state is very important. A lot of diagnostic information is processed in a very short amount of time (the initial global or gist view which occurs on less than 250 ms), so it is necessary to provide the best, most perceptually useful information in the initial default presentation. In part this is due to the desire to allow the user to make correct decisions with as little unnecessary image manipulation as possible without prolonging viewing times. In both radiology and pathology most image acquisition devices preprocess the images to improve overall appearance before sending them to the viewing workstation. If the preprocessing does what it intends, it can greatly reduce viewing times and the number of image manipulations (e.g., window/level or zooming) the user needs to carry out [75,76].

The default image does not preclude the availability and use of thumbnails, which is quite common in both pathology and radiology. In pathology the thumbnails are generally the various individual WSIs associated with a case; and in radiology they can be other images from the exam (e.g., CC and MLO views of the right and left breast for mammography), prior images, or images from other modalities (e.g., mammograms plus breast MRI). In both specialties, most viewing platforms typically have the thumbnails available on the left side of the display (sometimes across the top) with a large viewing window centered in the display area taking up most of the available space. The key from a perceptual perspective it to have the thumbnails big enough for the user to identify what they are so they can readily decide whether and when to bring them into full view, without having them so large as to interfere with the display of the images that are fully displayed. Ergonomically the goal is to have an interface that allows the user to easily access and move between the various thumbnails with as few mouse clicks as possible so as not to prolong viewing times unnecessarily or make navigation between images unwieldy.

An interesting difference between radiology and pathology is that most plain film (chest, bone mammography) exams are displayed on portrait mode displays while CT, MRI, and ultrasound are displayed on landscape displays; while in pathology landscape displays are used predominantly if not exclusively. To some extent the use of portrait mode for chest, bone and mammography has to do with the fact that the human body is generally taller than wider and thus the detectors are designed to match body dimensions to capture as much information as possible in a single acquisition. Actual film images preserved these dimensions and digital followed so it simply makes sense to view them on portrait displays preserving the natural aspect ratio. CT, MRI and ultrasound are not constrained by these dimensions as they are slices or views through the body (and physical dimensions are smaller than plain film images) so landscape displays are appropriate. Pathology slides are typically longer in one dimension than the other as well, but with light microscopy they are typically viewed left to right along the longer dimension which when moving to digital translates to landscape display. Orientation is also less of an issue in pathology than radiology (simply due to the scale of what is imaged) so landscape *vs.* portrait is far less of an issue. There are also no formal regulations (*i.e.*, from the FDA) regarding the types of and specifications for displays in pathology so the use of commercial displays rather than dedicated medical-grade displays is the norm and the majority of commercial displays use landscape as the default mode. With the advent of SmartPhones and mobile display devices that rotate images from portrait to landscape simply automatically as a function of how the display is held, getting the display to best fit the image is almost trivial.

## 6. Fatigue in the Digital Reading Environment

### 6.1. Why Study Fatigue?

Why are prolonged viewing times so important? On the one hand it impacts a department’s bottom line—how many cases are reviewed in a given period of time. At the human factors or individual level, however, the impact could be even more important. If it takes longer and takes more effort to review cases in an environment that has not been optimized, clinicians are likely to become fatigued and there is growing evidence that fatigue can impact diagnostic performance. For example, in one recent study [77], 40 radiologists and residents reviewed 60 bone exams, half with fractures, once before any clinical reading and once after a day of clinical reading. Reading time, visual accommodation (ability to maintain focus), subjective ratings of symptoms of fatigue and oculomotor strain, and diagnostic accuracy were measured. Diagnostic accuracy was reduced significantly by at least 4% after a day of clinical reading, and there was higher error in visual accommodation and greater subjective ratings of fatigue. An eye-position recording study on a sub-set of the images showed that late day reading resulted in longer viewing times and longer times to first fixate (land on and gaze at with high-resolution foveal vision) fractures whether or not they were eventually reported.

A second study confirmed the results, using CT (computed tomography) cases to determine if a modality/task that used dynamic (*i.e.*, stacks of multiple images that require scrolling through them) images rather than static plain film bone images [78]. Twenty-two radiologists and 22 residents searched CT chest sequences for a solitary pulmonary nodule before and after a day of clinical reading. Again, reading time, dark vergence (resting state of convergence of the eyes measured in the absence of stimuli/light), subjective ratings of symptoms of fatigue and oculomotor strain, and diagnostic accuracy were measured. Diagnostic accuracy was again reduced but at least 4%, dark vergence was significantly larger and more variable reflecting higher levels of visual strain, and subjective ratings of fatigue were higher.

### 6.2. Steps to Avoid Fatigue

There have yet to be any studies assessing the impact of fatigue on WSI interpretation in pathology, but it seems highly likely that if pathologists start spending the majority of their working hours in front of a computer display, fatigue effects will arise. There are a number of ways to try to avoid fatigue, but a few common sense ones are fairly easy to implement. First there is the 20-20-20 rule: every 20 min look away from the display at a point at least 20 feet away for at least 20 s. This reduces the temporary strain and induced myopia that the visual system experiences and actually helps with the second piece of advice—blink. Sitting in front of a computer for long hours leads to computer vision syndrome [79] and one of the common symptoms is dry eyes as a result of significantly reduced blink rates. Looking away from the display periodically, blinking, and using eye drops as needed can all help reduce the impact and strain of computer vision syndrome, potentially reducing fatigue and its negative diagnostic impact. 

Another easy step is to simply stand up for awhile while reading cases. For example, one study was conducted to determine if there are differences in physiologic vital signs (heart rate and blood pressure) of radiologists as a function of whether they read images while seated or standing up [80]. Five subjects had their blood pressure and heart rate measured while seated and standing reading cases in the normal clinical setting. For all three measures there was a statistically significant (*p* < 0.0001) difference between seated and standing measures with seated being lower than standing. The higher heart rate and blood pressure with standing suggests that the radiologists are more active in this position and thus potentially more attentive than while seated. There are a number of companies that sell computer tables that raise and lower quite easily, making this a very attractive option.

## 7. Mobile Devices and SmartPhones

### 7.1. Are Mobile Devices Adequate to Use Clinically?

Mobile devices such as iPads and SmartPhones are now being used in a wide variety of medical applications including imaging. The Food and Drug Administration (FDA) has already approved at least two viewers (mobile medical apps) for radiology (Resolution MD by Calgary Scientific, Inc., Calgary, AB, Canada and Mobile MIM by MIM Software, Cleveland, OH, USA). The approvals came with specifications about the types of images that can be viewed and the circumstances (including ambient light considerations) [81]. Both devices come with specifications that they are not intended to replace full workstations and are indicated for use only when there is no access to a workstation. In addition, since display performance can experience significant variations in luminance levels even between devices of the same model, labeling and safety features include steps to mitigate the risk of poor image display due to improper screen luminance or lighting conditions. The Mobile MIM device has an interactive contrast test in which a small part of the screen is a slightly different shade than the rest of the screen. If the user can identify and tap this portion of the screen, then the lighting conditions are not interfering with her ability to discern subtle differences in contrast.

The ultimate question of course is whether diagnoses made using mobile apps and devices are acceptable in terms of quality and under what circumstances can a primary read be given *vs.* a second opinion or over-read. One of the first studies to evaluate iPhones for teleradiology was looked at the diagnosis of acute cervico-dorsal spine trauma [82]. Two radiologists viewed a series of 75 CT cases of patients suspected of having cervico-dorsal spine fractures, once on the iPhone and once on a traditional workstation. Sensitivity and accuracy vertebral body fractures were 80% and 97%, and for posterior elements fractures was 75% and 98% for one reader and 50% and 97% for the other one. There were no significant differences between the iPhone and workstation.

Another early study assessed the interpretation of orthopedic (fracture) and CT (intracranial hemorrhage) images using a personal digital assistant (PDA) and an Apple iPod Touch compared to an off-the-shelf display monitor [83]. The PDA readings were actually significantly higher than with the monitor and although some of the other comparisons trended to significance they were all essentially equivalent for PDA and iPod *vs.* the monitor. Although there clearly needs to be more research, the results were promising for emergency use of mobile devices at least for these types of cases and findings.

A more recent study assessed interpretation of CT cases for pulmonary embolism using an iPad *vs.* a traditional PACS workstation [84]. The two readers interpreted 98% of the cases correctly (98% *vs.* 100% sensitivity; 98% *vs.* 96% specificity; 98% *vs.* 98% accuracy). Bhatia *et al.* also compared iPad (1,024 × 768 resolution) *vs.* standard PACS viewing (1,200 × 1,600 resolution LCD) for acute central nervous system events (emergency room generated CTA perfusion imaging, brain MRI, spine MRI), further confirming these results [85]. They found excellent intra-observer and inter-observer agreement for all three sets of cases and for radiologists and neurologists using the iPad. Other recent studies also indicate that iPads, SmartPhones and other mobile devices are useful for interpreting certain types of radiographic images [86,87]. The utility of iPads for reading CT and MRI images lies in the fact that iPad resolution is actually greater than what is actually required for these types of images. In general, it seems that SmartPhones and other mobile devices clearly have a role in teleradiology, but users should be aware of their limitations and understand the environmental and clinical conditions under which their use is appropriate [88,89,90].

As of now, there are no regulations for viewing WSI in terms of what devices and/or displays can or cannot be used. Although there are a number of options emerging, they should be carefully evaluated before being used for clinical reading. Mobile devices might be acceptable for second opinions or other types of consultations, but using them for primary is probably not recommended. In all likelihood however, as with radiology, mobile devices and associated viewing software apps will eventually be addressed by the FDA and there will be circumstances under which mobile displays will be appropriate and approved.

Even though there are no approved devices for pathology that does not mean there is no research on the topic. For example, a cell phone based contact microscopy platform called “*Contact Scope*” has been developed by one group [91]. It is a portable and compact microscope that can be installed on the camera unit of a cell phone. Using fiber-optic arrays and other technologies, a sequence of transmission images for the same sample are captured using the cell phone camera. The images are digitally fused based yielding a final microscopic image that is visualized through the phone screen. The system has been validated but not used clinically to date.

There are some more clinically oriented studies worth mentioning. For example, one recent study examined whether a tablet PC can be used to remotely diagnose dermatopathology cases [92]. The study used 93 cases diagnosed by conventional light microscopy that were imaged using a high-resolution video camera (Nikon DS-L2, version 4.4) mounted on a microscope. They were transmitted to an Apple iPAD2 (Apple Inc., Cupertino, CA, USA) via live streaming. Concordance with the original diagnosis and time to reach a diagnosis were recorded. The results showed that 92.5% of the cases were diagnosed in less than 5 s, with the average of 40.2 s. Of those diagnosed immediately, 98.8% of the tablet PC diagnoses were concordant with the original. They conclude that telepathology using a tablet PC may be a reliable and rapid method for diagnosing routine cases especially for institutions without the capital to purchase a dynamic robotic or a virtual slide system.

From a practical point of view for both teleradiology and telepathology the mobile devices need to be considered from a human factors perspective. Most current cell phones and tablet devices have very high resolution (“retinal”) displays that are quite close to what medical-grade displays offer. Additionally they have a lot of memory, desktop-quality computational power (64-bit processors), and high bandwidth (4G LTE) with some service providers. However, there are limitations as well. Although a number of phones are starting to have larger screens (almost the size of tablet devices), the majority are still quite small and thus even with the high resolution may not be all that well suited to reading radiographic and pathologic images. In the long-run it seems likely that there will need to be guidance from the FDA and Class I clearance may well be needed for many of these mobile viewer options.

### 7.2. Other Options on the Horizon

A more recent option is Google Glass which provides a high resolution display equivalent to a 25-inch high-definition screen from 8 feet away with a device that is simply embedded in eyeglass frames. There are however no studies to date on whether this type of device would be useful for medical imaging although one nice feature is that it has 12 GB of usable memory and can be synched with Google cloud storage making it feasible to access large images.

Mobile and smaller are obviously the major trends today, but there are efforts to go in the other direction as well. For example, Treanor *et al.* investigated the feasibility of a virtual reality Powerwall for viewing WSI [93]. They created a virtual reality microscope with an array of 28 20-inch high-resolution displays (total area 3 × 1.3 m) and total resolution of 53 million pixels. It was controlled using a computer game module with joystick and other common navigation tools. A user study indicated that it took only a few minutes for users to get used to it and they were able to perform a set of common interpretation tasks as quickly as they did with the microscope and with high confidence.

Large displays have also been considered for use in radiology. One study evaluated a large-screen rear-projection color display with resolution 1,920 × 1,080 and a 44-inch diagonal size [94]. For comparison it used the IBM 9 Mpixel color display (22-inch diagonal) set to a comparable resolution and maximum luminance. Diagnostic accuracy with a series of bone images with subtle fractures was to the traditional monitor, but viewing time was significantly shorter with the large display. The issue with large displays like these is the environment and space constraints. These large displays take up a lot of room and require the viewer to stand farther away typically than with smaller displays [94], making the design of a multi-user reading room a challenge and use of such displays in a typical office nearly impossible. The value of these larger displays however may be for use in conferencing situations or those in which multiple viewers need to see images simultaneously.

## 8. Conclusions

Where are teleradiology and telepathology headed with respect to human factors? They are certainly not going away, and it seems unlikely that traditional departments will disappear either. The future will likely be something new—a hybrid of on-site and remote practices as technologies change and the general practice of medicine changes. The current healthcare environment places a high premium on efficiency and increased access to quality care for all patients and teleradiology and telepathology represent very important ways that affordable healthcare will be available to all those that need it. The key is for users to be knowledgeable about the benefits and the costs (not just monetary) and to make informed decisions about how to provide services in the most efficient manner to provide quality care.

Similar to the trends we are seeing in personalized or tailored medicine, perhaps the idea of end-user profiling in medical imaging may be what lies ahead using human factors principles to design viewing systems and take into account the uniqueness of each individual end-user [95,96]. Medical imaging specialists differ from each other in a variety of ways, including their clinical experience, education, training, demographics, and specific job requirements. Other factors that contribute to inter-observer variability but are rarely studied include technology proclivity, personality, intelligence, emotional state, and sensory and motor skills. Perhaps we need to consider all of these when attempting to understand and optimize the digital reading environment and design and optimize systems (not just devices) that foster and facilitate efficient use of technologies that lead to quality interpretations of the wide variety of digital images radiologists and pathologists of the future will encounter. If we always keep in mind the cognitive, perceptual and physical capabilities (and limitations) of medical imagers as we design reading environments and technologies, we can improve patient care and maintain the health and well-being of the medical professionals.
